# **NEVi:** **N**egative **E**motional **Vi**deo dataset – categorizing stimulus intensity ratings based on valence and arousal

**DOI:** 10.1038/s41597-026-06870-8

**Published:** 2026-03-03

**Authors:** Hanne Schurig, Evelina Marie Stender, Julius Hennig, Michaela Ohme, Maria Seidel, Stefan Ehrlich

**Affiliations:** 1 Translational Developmental Neuroscience Section, Division of Psychological and Social Medicine and Developmental Neurosciences, Faculty of Medicine, Technische Universität Dresden, Dresden, Germany; 2 German Center for Child and Adolescent Health (DZKJ), partner site Dresden-Leipzig, Dresden, Germany

**Keywords:** Human behaviour, Psychology

## Abstract

We introduce **NEVi** (**N**egative **E**motional **Vi**deo dataset), a validated and standardized video dataset designed to evoke negative emotional responses. A total of 112 videos eliciting negative emotions were selected from established emotional video datasets. NEVi stands out by offering matched videos in two durations: short (1-sec) and long (5-sec), both individually extracted from the emotional video stimuli, taking into account the point of the highest intensity and the comprehensibility of the content. A total of 650 international, English-speaking volunteers evaluated the dataset by rating the stimuli on the dimensions of valence and arousal. Videos were categorized by intensity (low and high) based on these ratings. Particular care was taken to ensure the suitability of the video content for younger audiences, making it appropriate for use with adolescents. Results are provided as CSV files, containing participant demographics and ratings (overall and by gender) for valence and arousal. The data (CC-BY) are fully ‘open access’. Because stimuli come from third-party sources, we do not redistribute videos; instead, information is provided for reconstruction.

## Background & Summary

Using images to induce emotions is a common technique for eliciting and studying emotional responses. Despite their extensive use in emotion research, static images present notable limitations that challenge their ecological validity and effectiveness. Unlike dynamic stimuli such as films or videos, static pictures lack temporal continuity, and contextual embedding—features that are central to real-life emotional experiences^1,2^. As Gross and Levenson^3^ argue, emotions in everyday life are often evoked by dynamic audiovisual stimuli, making static images an oversimplification of natural emotional encounters. Dynamic facial expressions, for example, include transitional cues from one emotional state to another, which are absent in still images but are crucial for accurate emotion perception^4,5^. Static displays also fail to account for contextual influences—such as preceding expressions—which have been shown to significantly alter the interpretation of emotional content^6,7^. Furthermore, the reliance on static stimuli in social neuroscience has been criticized for limiting insights into interactive, multimodal, and temporally extended social processes^8,9^. This methodological limitation not only reduces emotional engagement but also raises concerns about the generalizability of laboratory findings to real-world social cognition. From a perceptual or ecological standpoint, judgments based on static images often diverge significantly from those based on dynamic presentations, questioning the validity of research based solely on photographic stimuli^10^. These findings collectively highlight the need to reconsider using static images. Dynamic, ecologically valid stimuli may provide a more accurate understanding of emotional and social processing.

Recently, researchers investigated how video stimuli can be employed to evoke emotions and have explored effective methods for measuring and analyzing these responses. Video stimuli have been shown to be highly efficient in evoking specific emotions such as happiness, sadness, relaxation, and fear, as confirmed by self-reports and expert evaluations^11^. In addition, compared with static images, their dynamic and multimodal characteristics not only make them more effective at provoking emotional responses^12^ but also enhance attention, increase physiological arousal, and improve memory retention, further underscoring their superiority for eliciting robust emotional reactions^13,14^. For example, neurophysiological studies have shown increased brain activation when subjects view video stimuli, indicating stronger emotional responses compared to static images^12^. While emotional impact may diminish with prolonged exposure when using pictorial stimuli^15^, video stimuli maintain affective processing over time^16^, providing an ecologically valid method to induce and assess emotional responses^17^. Moreover video stimuli, like static images, are adaptable to various needs and populations^18^.

Understanding the physiological basis of emotions involves examining both brain and peripheral processes. Emotional stimuli can significantly impact cognitive processes such as attention and memory, typically capturing attention more effectively and influencing memory based on the emotion’s valence and arousal^19^. Neural responses to emotional stimuli can be measured using Electroencephalography (EEG) and functional Magnetic Resonance Imaging (fMRI) techniques. EEG provides insights into temporal dynamics, while fMRI localizes brain regions involved in emotion processing^20^. Furthermore, video stimuli affect cardiac activity, influencing heart rate and blood pressure related to emotional intensity (arousal)^21^. Additionally, Galvanic Skin Response (GSR) measures skin conductance, which changes with emotional arousal and is often used alongside other physiological metrics to assess emotional responses^22^. Analyzing behavioral responses, such as analyzing facial expressions during and after video exposure, offer insights into immediate emotional reactions and subsequent regulatory processes^23^. Additionally, pupillary response and eye gaze measures reflect cognitive load and emotional arousal, with eye-tracking data revealing which aspects of the video capture attention^23^. Despite the findings mentioned above, so far, the majority of studies have used static images to investigate the effects on emotional, cognitive, and behavioral factors. This might partially be due to a limited availability of standardized emotional video stimuli^13^.

Given these considerations, we aimed to develop a new set of dynamic short videos, selected and rated for their affective content. Our goal was to create a set of video stimuli standardized in length and size, drawing from multiple categories (for example human interaction and nature) to elicit emotional responses using a variety of standardized video clips. Moreover, these videos were selected to be age-appropriate for adolescents and available in two different lengths: a short version (1 second [1-sec]) and a longer version (5 second [5-sec]) of the same video. The shorter version can be used as a priming stimulus, simplifying the investigation of how individuals manage and regulate their emotions or serving as a trigger for emotion regulation choice paradigms.

One significant advantage of our video stimulus set is its utility in investigating how individuals chose to regulate their emotional responses after exposure to emotionally evocative content. The inclusion of both short (1-sec) and longer (5-sec) versions of the same clips enables the short version to be used as a priming stimulus in emotion regulation tasks, allowing researchers to examine how individuals prepare for or engage in regulation strategies such as reappraisal or suppression. Emotional regulation, particularly reappraisal and suppression, plays a crucial role in mental health and well-being^24^. Reappraisal involves changing the way one thinks about a potentially emotion-eliciting event to alter its emotional impact, while suppression involves inhibiting the outward signs of emotion^25^.

In summary, this video stimuli set, indexed by valence, and arousal, is suitable for a wide range of research applications focusing on eliciting basic negative emotional responses in a controlled and reliable manner. Furthermore, it is ideal for studies on emotion regulation mechanisms.

## Methods

### Video stimuli selection

In our study, 113 previously published and validated video stimuli were selected from three sources, each offering a unique array of videos (see CSV file ‘Information_IDs_original_new.csv’). Our collection featured 41 videos from a dataset by Cowen & Keltner^26^, 39 from the LIRIS-ACCEDE Database^27^, and 33 from the Database of Emotional Videos from Ottawa (DEVO)^13^. Access to the DEVO dataset^13^ is available upon request from Patrick Davidson (patrick.davidson@uottawa.ca). The LIRIS-ACCEDE database^27^ is publicly available at https://liris-accede.ec-lyon.fr/. Access to the Cowen & Keltner emotion dataset^26^ can be requested via the following form: https://docs.google.com/forms/d/e/1FAIpQLScf9XVemSUWz6kUWySUdaQ5pxwqs8mugngrkBoLmX-3DMX1KA/viewform. The initial phase involved handpicking 152 videos from these sources. Selection criteria for creating a diverse collection prioritized video length, quality, and content. While ensuring the desired emotional reactivity, very violent scenes or clear depictions of abuse were excluded to also allow use in adolescent samples (assuming prior information and assent/consent). These videos were then uniformly edited to a length of 5 seconds. Next, a team of 22 individuals from our laboratory, including postdocs, PhD students, student assistants, and child and adolescent psychotherapists and psychiatrists, conducted an internal evaluation of these videos. Each rater independently evaluated 5-sec video clips using an online survey platform (Labvanced), which presented the videos in randomized order to reduce bias. The assessment focused on factors such as video quality, appropriateness for adolescent viewers, and clinical samples, as well as psychological parameters such as arousal and valence (as described in ‘Intensity Ratings’). Video quality and appropriateness were assessed using 9-point Likert scales (0 = not suitable/poor quality; 9 = highly suitable/excellent quality). Videos with average ratings equal to or below 5 on either dimension were excluded. Additionally, participants could provide qualitative comments on each video, which were also considered during the selection process. After rating each video, lab members were asked to mark the moment they perceived as having the highest emotional intensity. These time points were then averaged across raters to determine the peak intensity within each clip.

After this thorough screening, 39 videos were excluded due to unsuitability, leaving a refined set of 113 videos. Aligning with the methodology used by Sheppes *et al*.^28,29^, for each of these 113 video stimuli, both a 1-sec (short) and a 5-sec (long) version were created by extracting a 1-sec sequence out of the 5-sec video, resulting in a total of 226 stimuli. For the extraction of the 1-sec sequence, aspects such as the point with the highest intensity, as well as the semantic clarity and comprehensibility of the content were considered. This approach was used to provide a visual cue or priming stimulus, which is crucial for example investigating emotion regulation choice processes.

Following these preparatory steps, the final set of 226 video stimuli (113 long, 113 short) was stored separately as MP4 files, all formatted to a 16:9 aspect ratio and displayed at the same frame size (220 × 132 mm) to ensure uniformity across all videos. For cutting, trimming, and formatting the video stimuli to a uniform 16:9 aspect ratio and consistent frame size, we used the online video editing tool Online Video Cutter (https://online-video-cutter.com/de/). To validate our rating procedure, 40 test participants were recruited online using the crowdsourcing platform Prolific (www.prolific.com) for rating the two versions of the videos in terms of valence and arousal (same ratings as described in ‘Intensity Ratings’). This was accomplished via a partnership with the online platform Labvanced^30^, which was in charge of the recruitment and payment process. This stage was crucial for refining our evaluation script and formalizing the preregistration of the rating procedure (preregistration can be retrieved from 10.17605/OSF.IO/F5CWR). After preregistration, a large sample of participants were invited via the Prolific platform to rate the video dataset. Since the video stimulus 101 evoked primarily positive emotions and ratings (potentially due to difficulties understanding the content) in this larger test run, this stimulus was removed, leaving a final stimulus set of 112 videos in both long and short versions.

### Ethics

The study was conducted in accordance with the Declaration of Helsinki, and the Ethics Committee of TU Dresden approved its protocol (135042018). Prior to participation, each participant was thoroughly informed about the content and duration of the study and provided written voluntary, informed consent (electronic consent) for participation and for the public sharing of anonymized data in openly accessible research repositories in line with Open Science principles (e.g., OSF).This included a detailed description of the experiment’s content, a trigger warning regarding the presentation of negative video stimuli, and an emphasis on their right to decline participation or withdraw at any time; participants were also informed that, due to the high degree of anonymization, data cannot be retrospectively linked to individuals and therefore cannot be withdrawn or deleted after submission. Additionally, the confidentiality of their data was assured, and the data were recorded anonymously and stored in encrypted form for scientific evaluation. The consent form explicitly stated that only anonymized data would be shared and that no direct identifiers would be included in any publicly available materials. Moreover, participants were provided with a specific contact for any questions or concerns regarding the experiment or the rating procedure, ensuring they had continuous support and clear communication channels throughout the study.

### Participants

A total of 650 volunteers, aged 18 to 45, and registered prolific members were recruited for the main online experiment (described above under ‘Validation’). These are individuals who speak English and are resident outside Germany. The recruitment strategy was designed to achieve a balanced gender distribution, resulting in an equal split of approximately 50% women and 50% men. The participants in the study hailed from a variety of regions, with the majority coming from the United Kingdom (69.10%), Canada (10.86%), Australia (9.33%), the USA (7.64%), New Zealand (2.54%), and Ireland (0.50%). Data from 61 participants was excluded from the analysis (five failed manipulation checks, 19 displayed an overall average positive valence rating, 36 demonstrated exceptionally rapid reaction times during the rating process). This resulted in a final sample of 589 adults (including 299 females, 286 males, and 4 individuals with diverse gender identities) with an average age of 33.90 ± 7.40. Participants were compensated in accordance with Prolific’s hourly rate, ensuring they received fair payment for their 45 minutes of participation.

### Study procedure

After inclusion in the study, participants were informed about the content and duration of the study (45 minutes; viewing and rating the video sequences took 35 minutes), and were asked to keep the study in full-screen mode as well as to seek out a quiet environment without distractions. Using a customized link, the participants were then routed to the Labvanced^30^ web platform where the study was conducted. In the first part of the study, participants were asked to report their age, gender and country of residence. Additionally, participants were asked to provide information on current and past mental health issues, as well as the intake of psychotropic medication and eating disorder-related information (SCOFF questionnaire^31^ plus information on whether participants had a lifetime diagnosis of any eating disorder). Furthermore, they were asked to fill out the Brief Symptom Inventory–18^32^ and the Content-based Media Exposure Scale (C-ME2)^33^. The second part began with detailed instructions for the rating task and a brief definition of the concepts underlying the variables under assessment (valence and arousal; see ‘Intensity Rating’ for further details). These instructions and explanations were set up following the paper by Baralay *et al*.^13^, which provides further methodological details. Following the instructions, the task proceeded until completion. Within the task, 50 out of the 113 emotional video stimuli were presented in a completely randomized order. After the fixation cross was shown for 1.5 seconds, the short version of the video was shown. Subsequently, participants were asked to rate it on a 9-point scale in terms of valence (unhappy - happy) and arousal (relaxed - stimulated). After the rating, they were immediately shown the long version of the same video, which they were then also asked to rate. Once participants completed the rating on all scales, they could proceed to the next video stimulus. When the task was completed, participants were asked to give open feedback to assess their experience and highlight any encountered issues. All participants who successfully completed the study received monetary compensation.

### Intensity ratings

**Fig. 1:**
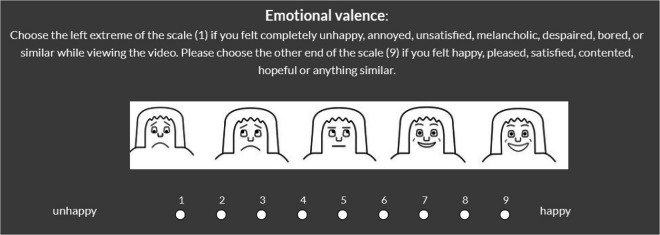
**Valence Scale.** Emotional valence rating scale used in the study. Participants rated their feelings on a 9-point scale ranging from unhappy (1 = negative affect) to happy (9 = positive affect), supported by a modified version of the Self-Assessment Manikin (SAM).

**Fig. 2:**
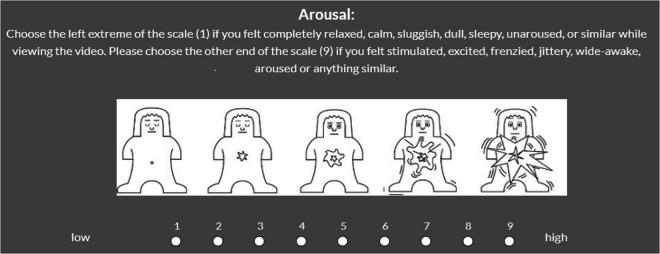
**Arousal Scale.** Arousal rating scale used in the study. Participants rated their level of arousal on a 9-point scale ranging from low (1 = relaxed, calm, unaroused) to high (9 = excited, jittery, highly aroused), supported by a modified version of the Self-Assessment Manikin (SAM).

Participant responses regarding the intensity of their emotional reactions were captured using a modified version of the Self-Assessment Manikin (SAM; UC3M4Safety SAM)^34^ (refer to Figs. 1, 2). This adaptation aimed to minimize gender bias in assessing emotional responses. The evaluation focused on two key aspects: valence (determining the positive or negative nature of the elicited emotion) and arousal (measuring the level of activation or intensity of the emotion experienced)^34^. Ratings of valence and arousal were paired with visual manakins as well as a short reminder text on the definition of the scale. Ratings were conducted on a 9-point scale ranging from feeling completely unhappy, annoyed, unsatisfied, melancholic, despaired, bored, or similar (1) to happy, pleased, satisfied, contented, hopeful or anything similar (9 [valence]) and respectively being completely relaxed, calm, sluggish, dull, sleepy, unaroused, or similar (1) to stimulated, excited, frenzied, jittery, wide-awake, aroused or anything similar (9 [arousal]) by cicking the specific bullet with the mouse. No time limit was given for the ratings.

## Data records

The NEVi dataset featured in this paper comprises both raw and processed data. These datasets are accessible via an OSF repository (10.17605/OSF.IO/F5CWR)^35^, which includes the necessary MATLAB files for data processing, executed using MATLAB R2021b^36^. To support data transparency and reproducibility, we have added a ‘README.txt’ file to the OSF repository that provides additional documentation not included in the manuscript, such as descriptions of variables, column headings, file names, and other relevant details.

### Data

The raw data is available in both compressed (‘00_raw_data.zip’) and uncompressed (‘00_raw_data’) formats. Each participant’s data is organized in individual folders, labelled with a session code (‘session_xxxxx’) generated by Labvanced. Within each folder, two.csv files can be found:

- **‘sessions.csv’**: Contains technical details such as the crowdsourcing code and the start and end times of each session.
- **‘trials.csv’**: Includes essential participant information, encompassing demographics, questionnaires, ratings, and feedback.

Additionally, the ‘02_logfiles’ and ‘02_logfiles.zip’ directories, available in both compressed and uncompressed formats, contain the ‘trials.csv’ files for each participant. These files are organized and summarized in files labelled ‘data_vpn_xxx.csv’, which denote the participant numbers..

- **csv files**: These files store raw data converted into comma-separated values format for ease of analysis.

### MATLAB library

The MATLAB library includes the scripts used for data processing, with functions designed to break down the process into a clear, step-by-step format. The available key files are:

- **‘AnalyzeData.m’**: main script containing the step-by-step process to generate the described processed data files. Please run this script if you want to analyse the data yourself.
- **‘transformLogfiles.m’**: file to extract needed.csv files from the raw data and organize them in the ‘02_logfiles’ directory.

Relevant MATLAB function files included in the library are:

- **‘readinlogfiles.m’**: Extracts information from the raw data files (‘02_logfiles’) and converts them into two structures containing demographic and questionnaire data, as well as the ratings of the video stimuli.
- **‘analyzeRegions.m’**: Analyses the origin data of participants.
- **‘saveFeedback’**: Saves participant feedback data in a csv file.
- **‘analyzeDemographics’**: Analyses the demographic data of participants.
- **‘outlier Detection’**: Detects and handles outliers in the dataset as described in the method section and preregistration.
- **‘analyzeMH’**: Analyses mental health-related data.
- **‘stimuliratings’**: Processes and analyses the ratings of the video stimuli.
- **‘stimuliSelection’**: Assists in the selection and categorization (high vs low) based on the intensity ratings of stimuli.

### Processed data

The processed and reported data include only the results from participants who met the criteria for data analysis (589 out of 650). Demographic information was gathered and stored in separate.csv files. The file ‘Demographics_region’ contains data on participants’ origins. Information on gender, age, psychological symptom scores (assessed using the short version of the Brief Symptom Inventory, BSI-18^32^), media consumption (measured via the Content-based Media Exposure Scale, C-ME^33^ – specifically, four questions from the physical violence domain), and eating habits (assessed using the SCOFF^31^, a screening tool for eating disorders) is available in two formats: mean values with standard deviations (‘Demographics_mean’) and median values with interquartile ranges (‘Demographics_median’). Additionally, normality distribution checks for these demographics are stored in the file ‘Demographics_normality’. Self-reported past (anamnestic) and current mental health conditions, as well as medication usage, are compiled in the ‘Mental_Health’ file. Participant feedback at the end of the rating process is summarized in the ‘Feedback’ file. Most importantly, the file ‘Results_rating_stimuli_both’ contains median and interquartile range for valence and arousal for both the 1-sec and 5-sec versions of the video stimuli. It also includes the total number of ratings per video, ranging from a minimum of 225 to a maximum of 317, with an average of 260.61 ratings per video stimulus (SD = 22.55). The stimulus ratings for female and male participants are stored separately in the files ‘Results_rating_stimuli_female’ and ‘Results_rating_stimuli_male’. Furthermore, several processed data.csv files are provided for female and male participants, as well as for both combined. These are indicated by the file suffixes (‘both’, ‘female’, ‘male’):

- **‘Stimuli_with_positive_valence’**: This file lists the stimuli with an overall positive valence that need to be excluded from the dataset. It contains only one stimulus (stimuli_101.mp4).
- **‘End_high_stimuli’**: This file includes the 40 high-intensity stimuli along with their additional ratings and information.
- **‘End_low_stimuli’**: This file includes the 40 low-intensity stimuli along with their additional ratings and information.
- **‘End_middle_stimuli’**: This file includes the remaining stimuli that are not classified as high or low intensity.
- **‘End_highvslow_stimuli_mean_diff’**: This file contains the mean values and standard deviations, comparing the ratings between high and low-intensity video stimuli.

This detailed organization ensures that the dataset is accessible and comprehensible for further analysis.

### Stimuli

**Fig. 3:**
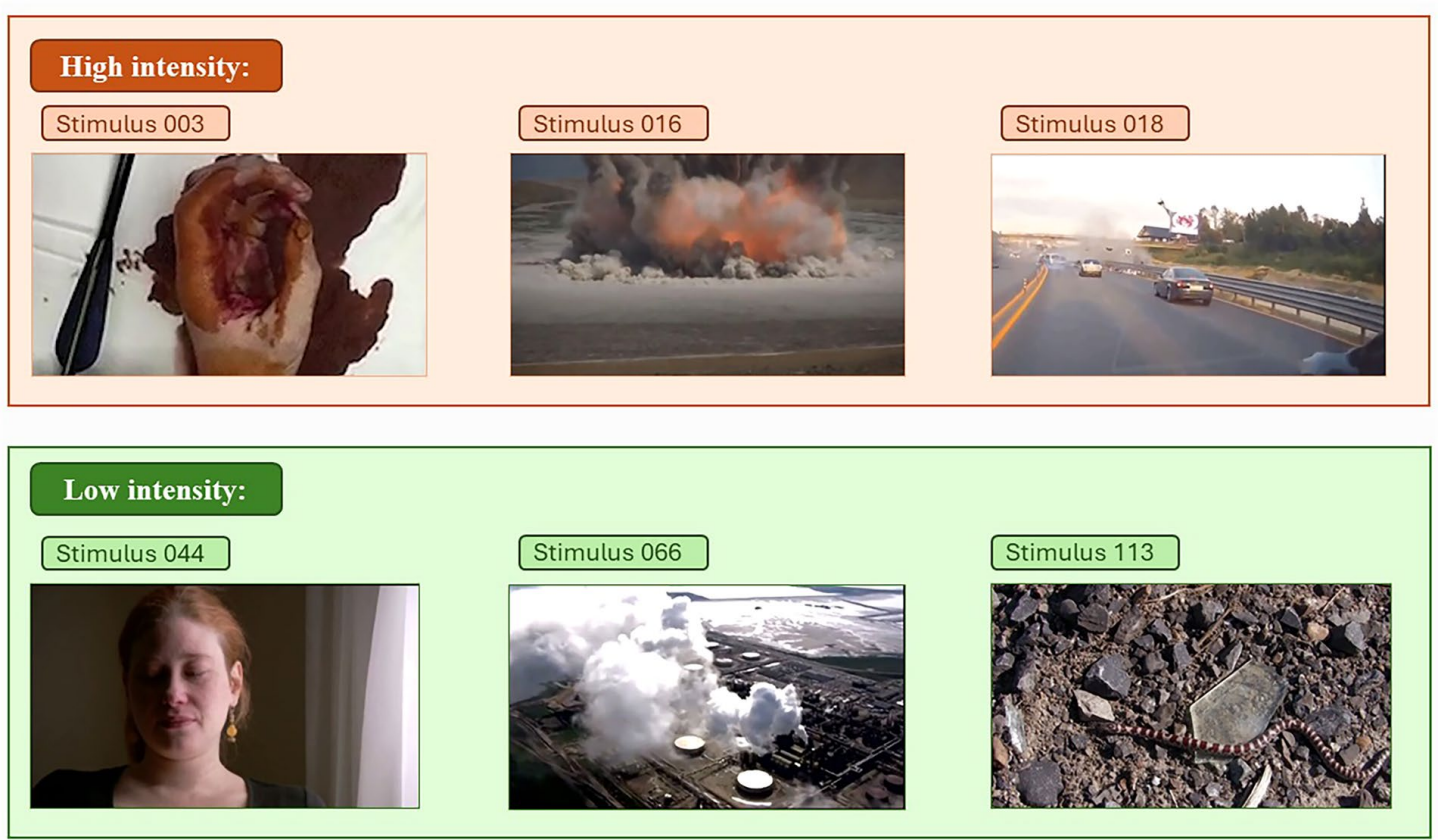
**Example stimuli from the NEVi dataset.** The upper panel presents three negative stimuli (Stimuli 003 - “injury”, 016 - “explosion”, and 018 – “car crash”) from the high-intensity group, and the lower panel shows three negative stimuli (Stimuli 044 - “crying”, 066 – “pollution”, and 100 – “plastic ocean”) from the low-intensity group. Together, these examples depict the range of emotional intensity and content included in the dataset.

The NEVi video stimuli (refer to Fig. 3) are derived from third-party video sources (Cowen & Keltner^26^, DEVO^13^, and LIRIS-ACCEDE^27^) and are therefore not redistributed directly with this publication; instead, the OSF repository provides the edit-point information needed for reconstruction from the original datasets (‘Information_videos_edit_points.csv’), along with detailed instructions in the video stimuli selection section. For the extraction of video segments and the subsequent formatting of all stimuli to a standardized 16:9 aspect ratio and identical frame dimensions, we used the online video editing tool Online Video Cutter (https://online-video-cutter.com/de/). After independently obtaining the raw video materials under their respective licenses, users can follow these step-by-step instructions to apply the provided edit points and cut the original videos accordingly, thereby reproducing the exact NEVi stimulus set used in the present study. This procedure ensures full transparency and reproducibility while respecting the licensing restrictions of the original video sources. The stimuli share several common features. All videos were standardized to a 16:9 aspect ratio and presented in a uniform frame size during the experiment, although their native resolutions varied. All clips are in color. To ensure consistency, videos were presented without sound, even when the original source contained audio. Any potentially distracting elements — such as subtitles, timestamps, or other textual overlays — were removed. The stimuli were selected from three sources (see above) each of which integrates material from a broad range of origins, including independent films, documentaries, open-access research databases^26^, and publicly available online platforms (for example YouTube and Vimeo)^13^. Mainstream Hollywood productions were intentionally excluded to avoid a high likelihood of familiarity with the stimuli^13^. Owing to these diverse origins, the clips vary in recording style: some were filmed with professional equipment, while others were captured using handheld cameras, dashcams, or mobile phones. The content spans a wide spectrum, from unscripted footage of real-world events (for example sporting activities or environmental incidents) to scripted scenes designed to evoke negative affect (for example interpersonal conflict or threat). This diversity enhances ecological validity by encompassing a broad range of negative emotional contexts, while systematic categorization and editing procedures ensure comparability across stimuli.

The videos are organized into several directories. The ‘01_all’ directory contains all 224 stimuli, which are further divided into two subfolders for the 5-sec and 1-sec versions. Additionally, the stimuli are sorted by intensity, with 40 high-intensity and 40 low-intensity videos for easier handling and use (if the researcher wishes to differentiate between the two categories – though this is not obligatory). The categorization is provided for all participants (categorized_all_participants) and separately for female (categorized_female_participants) and male participants (categorized_male_participants) to facilitate use across different target groups. In the ‘01_stimuli_information’ directory, you can find comprehensive details about the stimuli. This includes the file ‘Information_IDs_original_new.csv’, which maps the naming of the NEVi stimuli to their corresponding stimuli from the original databases. Additionally, the ‘content.csv’ file provides information on the content of the video stimuli, categorized by people, faces, nature, animals, and vehicles. This categorization ensures that the high- and low-intensity groups are content-matched, with no more than a two-item difference between them (refer Table 1 for more details). Moreover, the directory contains the ‘Information_videos_edit_points.csv’ file, which details the exact points where the 1-sec and 5-sec sequences were extracted from the original videos. This provides clear information about how the versions of the videos were generated. These files collectively offer a thorough overview of the stimuli, their content, and the methodology used to create the different versions, ensuring transparency and aiding further research. Additionally, the video stimuli were divided into high- (40 stimuli), middle- (32 stimuli), and low-intensity (40 stimuli) stimuli depending on their rating (Results_rating_stimuli_intensity).

**Table 1:** Distribution of the individual videos across the categories.

Content	Number of videos (n = 112)	High intensity videos n = 40 (all genders)	Low intensity videos n = 40 (all genders)
People	84	33	32
Faces	21	4	6
Nature	8	2	2
Animals	16	6	4
Vehicles	11	3	3

**Fig. 4:**
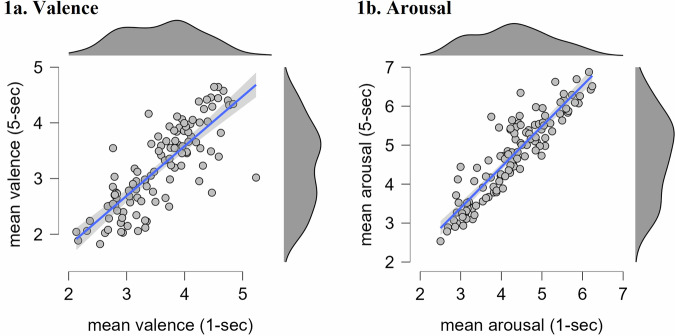
**Distributions.** Descriptive plots showing the distributions of mean ratings for **(1a)** valence and **(1b)** arousal across the 1-sec and 5-sec versions of each video stimulus. The scatter plots illustrate the relationship between the 1-sec and 5-sec ratings. The density plots above each panel display the distribution of values for each measure. *Note*. Shaded areas around the regression lines indicate 95% confidence intervals. N = 112 per video version (1-second and 5-second). Plots were generated using JASP (version 0.19.3.0).

The rating for each video can be derived from the ‘Results_rating_stimuli_both.csv’ or in Supplementary Table 1, which provides detailed information including content descriptions and the number of raters. All results are presented as mean ± standard deviation (see Table 2). The mean arousal rating (1 = relaxed, 9 = stimulated) for the 1-sec videos categorized as high intensity videos was 5.21 ± 0.55, the mean valence (1 = unhappy, 9 = happy) was 3.14 ± 0.57. Regarding the low intensity 1-sec videos, mean arousal rating was 3.65 ± 0.68, the mean valence was 3.84 ± 0.61. The mean arousal rating for the 5-sec videos categorized as high intensity videos was 5.61 ± 0.6, the mean valence was 2.87 ± 0.64. Regarding the low intensity 5-sec videos, mean arousal rating was at 4.05 ± 0.89, the mean valence was 3.46 ± 0.78. The rating distributions are further illustrated in Fig. 4, which displays the mean valence and mean arousal ratings for both the 1-sec and 5-sec video versions, along with the range and density of values for each measure.

**Table 2:** Mean and standard deviation across ratings for different video categories.

Video intensity	Valence (M ± SD)			Arousal (M ± SD)		
	All	Male	Female	All	Male	Female
**1-sec (short)**						
**High**	**3.14** ± *0.57*	**3.35** ± *0.58*	**3.02** ± *0.63*	**5.21** ± *0.55*	**5.40** ± *0.45*	**5.15** ± *0.53*
**Low**	**3.84** ± *0.61*	**3.86** ± *0.61*	**3.91** ± *0.59*	**3.65** ± *0.68*	**3.67** ± *0.67*	**3.50** ± *0.62*
**5-sec (long)**						
**High**	**2.87** ± *0.64*	**3.15** ± *0.68*	**2.73** ± 0.69	**5.61** ± 0.60	**5.73** ± 0.54	**5.62** ± *0.59*
**Low**	**3.46** ± *0.78*	**3.54** ± *0.7*	**3.54** ± *0.69*	**4.05** ± *0.89*	**4.01** ± *0.79*	**3.90** ± *0.84*

## Technical Validation

### Quality of data

To ensure transparency and reproducibility of our data, we carefully outlined the rating procedures and exclusion criteria in a preregistration^35^. The rating and validation study included six manipulation checks, with two of them being part of the demographic and questionnaire sections. For these checks, participants were instructed to choose a specific answer to particular questions, such as being directed to select ‘extremely’ in response to a certain prompt. In addition, during the rating process, we interspersed four other manipulation checks at regular intervals. These required participants to tick a specific answer on an easy-to-difficult rating scale at four specific times – during trials 11, 21, 31, and 41. We also asked participants to encapsulate the content of the video in a single word on five separate occasions – during trials 7, 17, 27, 37, and 47. These measures were put in place to guarantee the reliability and validity of the participants’ ratings. Participants who incorrectly answered three or more of these manipulation checks were excluded from the study. Additionally, participants were excluded if their mean valence ratings were neutral or positive (given that all our videos had a negative content), or if their average response time — from the onset of the rating page until they clicked to view the next clip (excluding clip duration) — was under six seconds, suggesting a hasty, inattentive response. These strict rules where established to preserve the quality of our data and research outcomes.

### Statistical analyses

**Fig. 5:**
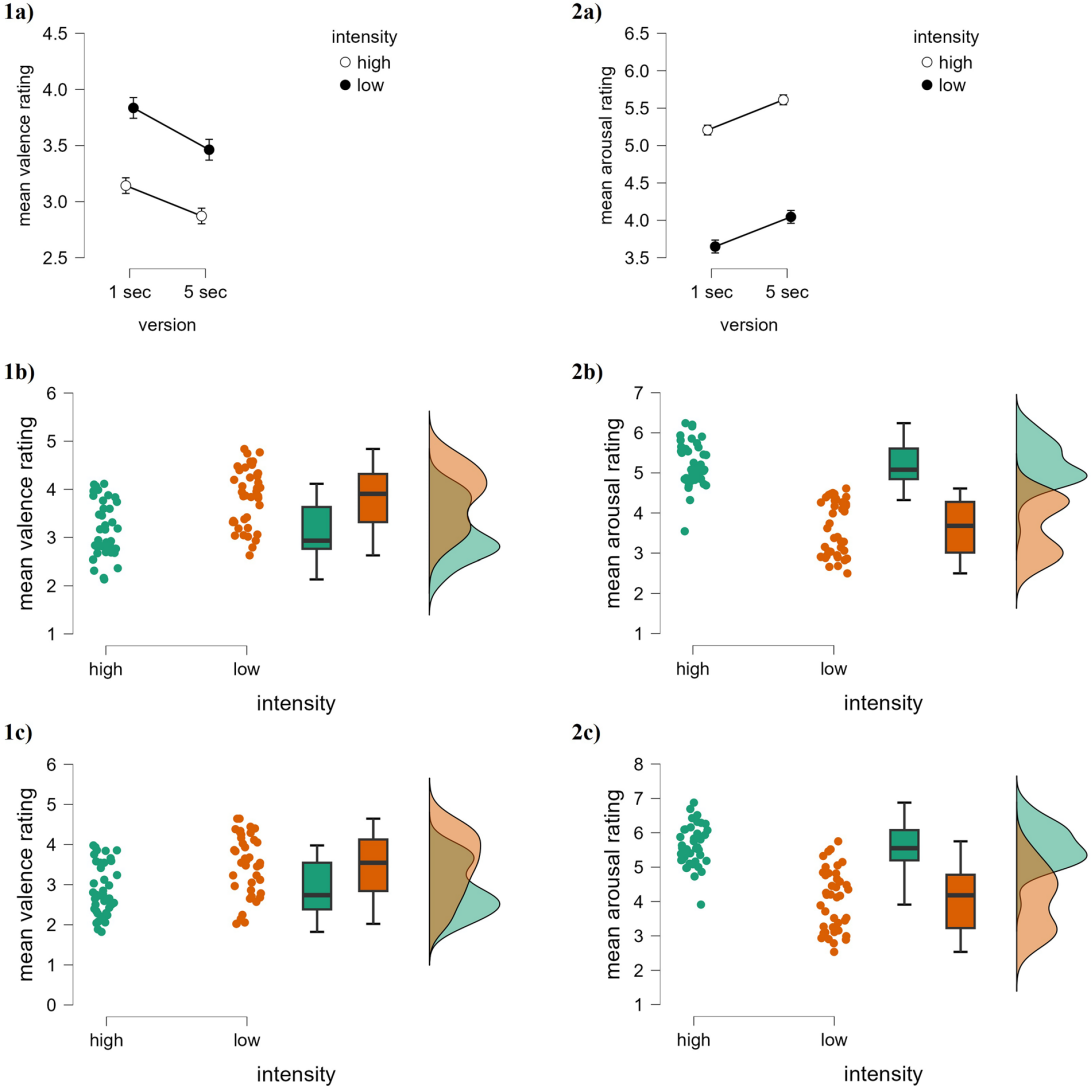
**Statistical analyses.** Descriptive plots and visualization of Bayesian 2 × 2 repeated-measures ANOVAs. **(1a,2a)** Descriptive plots of mean valence **(1a)** and mean arousal **(2a)** ratings for both video versions and intensity conditions. **(1b,1c)** Raincloud plots showing individual data points, boxplots, and distributions for 1-sec **(1b)** and 5-sec **(1c)** versions, comparing mean valence ratings between high- and low-intensity stimuli. **(2b,2c)** Raincloud plots showing individual data points, boxplots, and distributions for 1-sec **(2b)** and 5-sec **(2c)** versions, comparing mean arousal ratings between high- and low-intensity stimuli. *Note.* Error bars around means indicate 95% credible intervals. N = 40 per intensity group (high vs. low). Plots were generated with JASP (version 0.19.3.0).

As a first step, we provided an overview of the ratings for each video clip (see Supplementary Table 1). Furthermore, we investigated whether shortened 1-sec video clips can effectively convey emotional content and whether emotional responses differ significantly between high- and low-intensity stimuli, as measured by valence and arousal. For this, we conducted a series of Bayesian 2 × 2 repeated-measures (RM-)ANOVAs using JASP (version 0.19.3.0) examining the main effects of video duration (1-sec vs. 5-sec) and emotional intensity (low vs. high), as well as their interaction, on participants’ valence and arousal ratings. A visualization of the results is provided in Fig. 5.

#### Valence ratings

There was very strong evidence for a main effect of video version on valence ratings, as shown by a high Bayes inclusion factor (BF_incl_ = 3.643 × 10⁸), with 1-sec videos resulting in higher valence ratings compared to 5-sec videos. This difference was confirmed by a post-hoc Bayesian paired sample t-test in both intensity categories (high intensity: BF_10_ = 8563.1, low intensity: BF_10_ = 15630.5). The intensity factor also showed strong evidence for a negative main effect on valence, with high-intensity videos yielding lower valence ratings (BF_incl_ = 629.7), reflecting more averse emotional responses. This difference was confirmed by a post-hoc Bayesian independent sample t-test (1-sec: BF_10_ = 10800.5, 5-sec: BF_10_ = 64.1). There was no evidence for an interaction effect between version and intensity (BF_incl = _1.652), suggesting that their effects on valence were largely independent.

#### Arousal ratings

There was also strong evidence for a main effect of version (BF_incl_ = 2.639 × 10¹³), with 1-sec videos resulting in lower arousal ratings than 5-sec videos. This difference was confirmed by a post-hoc Bayesian paired sample t-test (high intensity: BF_10_ = 1.228 × 10^8^, low intensity: BF_10_ = 194552.7). The intensity factor showed equally strong evidence for a positive main effect (BF_incl_ = 9.925 × 10¹²), with high-intensity videos producing higher arousal. This difference was confirmed by a post-hoc Bayesian independent sample t-test (1-sec: BF_10_ = 7.475 × 10^14^, 5-sec: BF_10_ = 1.350 × 10^11^). Once again, there was no evidence for an interaction effect between version and intensity (BF_incl_ = 0.913), indicating minimal interdependence between the two factors.

Despite the fact that 1-sec videos elicited less intense valence and arousal ratings than their 5-sec counterparts, they preserved the overall emotional direction of responses. High-intensity clips were consistently rated as more negative and more arousing, regardless of video duration. This pattern consistency suggests that shortened stimuli can still convey the core emotional message, making them suitable for use as priming stimuli in experimental paradigms. This is further supported by the strong and highly significant correlations observed between the 1-second and 5-second videos for both valence (ρ = .80, p < .001) and arousal (ρ = .89, p < .001).

### Validation of emotional response data using self-report measures

To assess the quality and construct validity of the dataset, we performed correlational analyses to evaluate whether participants’ emotional ratings aligned with theoretically expected patterns based on individual difference measures.

First, we examined correlations between participants’ average arousal ratings and their scores on the Global Severity Index (GSI) from the BSI-18^32^, which assesses psychological symptomatology across somatization, depression, and anxiety. Prior research^37–39^ has shown that individuals with higher psychological distress tend to exhibit heightened arousal when exposed to emotional stimuli. Consistent with these findings, we observed small significant positive correlations between GSI scores and average arousal ratings for both the 1-sec and 5-sec video conditions (r = 0.12, p < .01 for both), suggesting that participants with higher symptom levels perceived the emotional content as more arousing.

Next, we investigated whether consumption of violence in media influenced participants’ average valence ratings. To this end, we used four items from the physical violence domain of the C-ME2^33^, which gauges the frequency of exposure to violent media content. Based on previous findings^40,41^, we hypothesized that greater media violence exposure would be associated with reduced emotional responsiveness, reflected in more positive valence ratings, thereby indicating a desensitization effect. Supporting this hypothesis, correlation analyses revealed small but significant positive associations between media consumption and valence ratings for both 1-sec (r = 0.17, p < .01) and 5-sec (r = 0.16, p < .01) stimuli.

Together, these analyses serve as internal validation checks, demonstrating that participants’ emotional ratings reflect relevant psychological and behavioral traits. These results support the validity of the dataset, reinforcing its potential for future research applications.

### Descriptive comparison to IAPS ratings

In a previous study by Sheppes *et al*.^29^, a total of 80 negative images from the International Affective Picture System (IAPS)^42^, which uses the same 9-point Likert scale for arousal (1 = low, 9 = high) and valence (1 = very unpleasant, 9 = very pleasant), were divided into two intensity categories based on their normative ratings. The high-intensity group (n = 40) included images with mean arousal of 6.45 and mean valence of 1.87, while the low-intensity group (n = 40) had mean arousal of 5.00 and mean valence of 3.39. Many of the high-intensity IAPS images used in that study featured highly graphic content, including depictions of death, serious injury, medical trauma, and violence (for example images 3000, 3010, 3061, 3102, 9253). Such imagery, while effective for eliciting strong emotional reactions, may be unsuitable for adolescents or individuals with mental disorders or mental health problems, due to its potentially distressing nature.

In contrast, the NEVi video stimuli offer dynamic content chosen to elicit negative emotions while remaining within ethically appropriate boundaries. The content was selected to be naturalistic and suitable for younger participants, without resorting to extreme or disturbing scenes. For the high-intensity NEVi 5-sec videos, mean arousal ratings were 5.61, while corresponding valence ratings 2.87, respectively. The low-intensity 5-sec videos received arousal ratings of 4.05, with valence ratings of 3.46, respectively. Although NEVi stimuli tend to elicit slightly less extreme ratings than the most aversive IAPS images from Sheppes *et al*.^29^, the relative differences between low- and high-intensity stimuli are comparable. This suggests that NEVi can be a viable alternative for studies requiring emotional differentiation—without the ethical and clinical concerns associated with more graphic material—making it particularly well-suited for use with youth populations and individuals with increased psychological vulnerability.

## Usage Notes

NEVi, a validated dataset of 112 videos evoking negative emotions, is available for use in a variety of studies. Both the short and long versions of the video stimuli can be used together or separately, although the number of available videos is limited (112). As this dataset is indexed based on valence and arousal it is suitable for research on emotion induction and emotion regulation paradigms. Naturally the 5-sec video versions can also be used on their own in any other experimental study aiming to elicit an affective response in a controlled, reliable manner.

The dataset can also be valuable in several additional fields of psychology:

- In cognitive studies it can be used to examine perception, visual attention, and emotional memory.
- In social psychology it can help explore topics such as morality, responsibility, and empathy.
- In clinical samples it can be employed for emotional training, memory training, reality monitoring, and memory distortion studies.

Future research might consider additional factors such as luminance, the role of perspective-taking, and age-related differences. At the same time, several limitations should be noted. First, the videos have no audio track, as not all original stimuli contained sound. The absence of audio information may have influenced participants’ emotional experiences, given the well-established role of sound in shaping emotional responses. Second, the dataset includes only negative stimuli, resulting in an imbalance in the valence dimension. This was a deliberate design choice, grounded in clinical and theoretical relevance: negative affect is central to many mental health conditions such as depression, anxiety, and Post-traumatic stress disorder. Difficulties in regulating negative emotions are core features of these disorders. By focusing on negative valence, the dataset is intended as a resource for research on negative emotion regulation and psychopathology in clinical psychology and affective neuroscience. Nevertheless, this asymmetry limits the dataset’s breadth, and future work could extend it by incorporating positive or neutral stimuli. Researchers who wish to do so may also supplement the current dataset with other available resources. Third, the study was conducted in English, which introduces a language bias and limits applicability to English-speaking participants. Fourth, the sample was drawn exclusively from the Prolific platform, which may restrict the generalizability of the findings to populations beyond this participant pool. Finally, all data were based on self-report measures, which are inherently susceptible to biases such as social desirability and inaccurate recall.

## Supplementary information

Supplementary Table 1.

## Data Availability

The data supporting this study are openly available in the Open Science Framework (OSF) repository^35^ at 10.17605/OSF.IO/F5CWR under a Creative Commons Attribution 4.0 International License (CC BY 4.0). The stimulus video clips are not redistributed directly with this dataset because they originate from third-party sources (Cowen & Keltner^26^, DEVO^13^, and LIRIS-ACCEDE database^27^). They are subject to their respective licenses but are easy to obtain at no cost (for research purposes). Instead, we provide the edit-point information and additional instructions required for reconstructing the NEVi stimuli after obtaining the original video datasets, available in the OSF repository as ‘Information_videos_edit_points.csv’.

## Code availability

The NEVi dataset featured in this paper comprises both raw and processed data. These datasets are accessible via an OSF repository^35^, which includes the necessary MATLAB files for data processing, executed using MATLAB R2021b. A more detailed description of the MATLAB files, the functions, and the data-processing workflow can be found in the paper’s “Data Records” section.
