# Supplementary material for: NEVi: Negative Emotional Video dataset – categorizing stimulus intensity ratings based on valence and arousal
Source: Sci Data. 2026 Mar 3;13:322. doi: 10.1038/s41597-026-06870-8 (PMC12960829; doi:10.1038/s41597-026-06870-8)
Supplement: Supplementary file 1 — Supplementary Table 1. [file 41597_2026_6870_MOESM1_ESM.pdf]

Supplementary Table

Supplementary Table 1. Mean and standard deviation of all ratings for each video, including a brief description of the video content and the total number of ratings received. *Videos were excluded due to a positive mean valence rating.

id	content	Ratings (n)	Valence (M ± SD)		Arousal (M ± SD)	
			1-sec	5-sec	1-sec	5-sec
1	**Carcrash**	240	**3.83** *± 1.33*	**3.85** *±1.51*	**4.87** *± 2.34*	**5.08** *±2.30*
2	**Height**	250	**3.59** *± 1.67*	**3.41** *±1.74*	**5.93** *± 2.38*	**6.26** *±3.24*
3	**Injury**	225	**2.76** *± 1.31*	**3.54** *±1.82*	**4.99** ± 2.31	**4.72** *±2.32*
4	**Carcrash**	228	**3.73** *±1.27*	**2.49** *±1.35*	**4.47** *± 2.22*	**6.34** *±2.40*
5	**Carcrash**	262	**2.16** *± 1.28*	**1.88** *±1.16*	**5.90** *± 2.48*	**6.07** *±2.50*
6	**War**	257	**3.01** *± 1.37*	**2.14** *±1.29*	**4.35** *± 2.12*	**5.14** *±2.48*
7	**Carnivorous plant**	243	**3.65** *± 1.70*	**3.32** *±1.72*	**4.29** *± 2.14*	**4.48** *±2.31*
8	**Fight**	251	**3.37** *± 1.36*	**4.16** *±2.14*	**4.15** *± 2.01*	**4.84** *±2.06*
9	**Tornado**	229	**3.86** *± 1.71*	**3.84** *±1.92*	**5.81** *± 2.23*	**6.09** *±2.27*
10	**Injury**	228	**4.46** *± 1.64*	**2.74** *±1.71*	**3.94** *± 2.21*	**5.45** *±2.47*
11	**War**	256	**3.91** *± 1.59*	**3.91** *±1.57*	**3.81** *± 1.98*	**3.78** *±2.04*
12	**Shark attack**	243	**3.98** *± 1.49*	**3.85** *±1.71*	**5.07** *± 2.16*	**5.75** *±2.30*

id	content	Ratings (n)	Valence (M ± SD)		Arousal (M ± SD)	
			1-sec	5-sec	1-sec	5-sec
13	**Carcrash**	252	**3.41** *± 1.35*	**3.05** *±1.49*	**4.26** *± 2.13*	**4.57** *±2.44*
14	**Wedding dying**	250	**3.06** *± 1.60*	**2.57** *±1.56*	**3.74** *± 2.17*	**4.16** *±2.37*
15	**Knife game**	232	**3.59** *± 1.54*	**3.59** *±1.75*	**5.63** ± 2.33	**5.83** *±2.50*
16	**Explosion**	248	**3.73** *± 1.76*	**3.93** *±1.84*	**5.08** *± 2.48*	**4.97** *±2.46*
17	**Shitting animal**	254	**3.16** *± 1.82*	**2.95** *±1.87*	**3.80** *± 2.37*	**3.92** *±2.54*
18	**Carcrash**	235	**2.89** *± 1.54*	**2.20** *±1.37*	**5.54** *± 2.31*	**6.15** *±2.39*
19	**Dangerous animal**	246	**4.45** *± 1.32*	**4.45** *±1.81*	**4.42** *± 2.20*	**5.56** *±2.27*
20	**Drowned person**	239	**3.18** *± 1.42*	**2.65** *±1.42*	**5,74** ± 2.38	**6.28** *±2.39*
21	**Shooting**	258	**3.16** *± 1.47*	**2.80** *±1.43*	**4.62** *± 2.25*	**5.00** *±2.38*
22	**Heart attack**	264	**4.31** *± 1.24*	**3.23** *±1.53*	**2.92** *± 1.74*	**4.12** *±2.24*
23	**Spiders**	242	**2.13** *± 1.35*	**2.04** *±1.41*	**6.20** *± 2.59*	**6.42** *±2.67*
24	**Heart attack**	246	**3.19** *± 1.47*	**2.05** ±1.22	**4.35** *± 2.20*	**5.45** *±2.47*
25	**Bear**	255	**4.25** *± 2.26*	**2.95** *±2.06*	**4.02** *± 2.13*	**4.71** *±2.36*
26	**Fire**	231	**3.68** ± 1.54	**3.67** *±1.72*	**4.21** *± 2.16*	**4.29** *±2.24*

id	content	Ratings (n)	Valence (M ± SD)		Arousal (M ± SD)	
			1-sec	5-sec	1-sec	5-sec
27	**Falling**	242	**2.68** *± 1.46*	**2.50** *±1.71*	**6.23** *± 2.31*	**6.51** *±2.43*
28	**Slaughter**	257	**2.93** *± 1.61*	**2.02** *±1.40*	**4.45** *± 2.32*	**5.75** *±2.56*
29	**Snowcrash**	233	**4.09** *± 1.36*	**3.97** *±1.59*	**4.80** *± 2.21*	**5.21** *±2.26*
30	**Alligator attack**	243	**3.87** *± 1.54*	**3.54** *±1.67*	**5.45** *± 2.42*	**6.09** *±2.30*
31	**Heigth**	247	**4.11** *± 1.63*	**3.75** *±1.85*	**5.85** *± 2.35*	**6.69** *±2.20*
32	**Shark attack**	247	**4.06** *± 1.70*	**3.65** *±1.87*	**5.44** ± 2.34	**5.80** *±2.45*
33	**Tornado**	236	**3.45** *± 1.44*	**2.76** *±1.54*	**4.85** *± 2.30*	**5.97** *±2.33*
34	**Pimpel**	234	**2.76** *± 1.89*	**2.85** *±2.07*	**5.50** *± 2.47*	**5.63** *±2.61*
35	**Dead person**	244	**2.94** *± 1.36*	**2.39** *±1.28*	**3.54** *± 2.10*	**3.90** *±2.21*
36	**Explosion**	238	**3.81** *± 1.61*	**3.86** *±1.71*	**4.30** *± 2.13*	**4.45** *±2.22*
37	**Fall in fire**	234	**3.49** *± 1.86*	**2.58** *±1.86*	**5.32** *± 2.38*	**6.62** *±2.36*
38	**Colliding cars**	251	**3.82** *± 1.35*	**3.19** *±1.68*	**4.22** *± 2.19*	**5.39** *±2.37*
39	**Heigh Building**	251	**3.76** *± 1.65*	**3.23** *±1.84*	**6.15** *± 2.44*	**6.87** *±2.36*
40	**Injury fight**	237	**3.54** *± 1.59*	**3.75** *±1.80*	**4.57** *± 2.28*	**4.71** *±2.42*

id	content	Ratings (n)	Valence (M ± SD)		Arousal (M ± SD)	
			1-sec	5-sec	1-sec	5-sec
41	**Burning**	241	**2.81** *± 1.44*	**2.74** *±1.42*	**5.08** *± 2.30*	**5.18** *±2.34*
42	**Crying**	283	**3.75** *± 1.28*	**3.55** *±1.41*	**3.11** *± 1.81*	**3.24** *±1.95*
43	**Detonation**	258	**3.25** *± 1.63*	**2.59** *±1.47*	**4.82** *± 2.44*	**5.51** *±2.36*
44	**Crying**	270	**4.06** *± 1.34*	**3.83** *±1.39*	**2.68** *± 1.67*	**2.88** *±1.75*
45	**Argument**	266	**4.74** *± 1.08*	**4.40** *±1.18*	**2.90** *± 1.80*	**3.27** *±1.98*
46	**Fight**	259	**2.80** *± 1.28*	**2.51** *±1.29*	**4.82** *± 2.23*	**5.10** *±2.36*
47	**Injury**	231	**2.54** *± 1.49*	**1.82** *±1.24*	**5.53** *± 2.42*	**6.31** *±2.55*
48	**Crying**	259	**4.03** *± 1.35*	**3.45** *±1.38*	**3.20** *± 1.97*	**3.54** *±1.98*
49	**Desperate**	237	**3.55** *± 1.26*	**3.49** *±1.28*	**3.68** *± 2.02*	**3.66** *±2.06*
50	**Stabbing**	257	**2.82** *± 1.48*	**2.64** *±1.46*	**5.25** *± 2.38*	**5.35** *±2.47*
51	**Illness**	262	**4.58** *± 1.12*	**3.39** *±1.36*	**2.99** *± 1.83*	**4.44** *±2.15*
52	**Dead body**	266	**4.03** *± 1.29*	**3.47** *±1.44*	**3.62** *± 1.90*	**4.19** *±2.07*
53	**Fight**	255	**3.95** *± 1.36*	**3.45** *±1.54*	**4.26** *± 2.14*	**4.81** *±2.28*
54	**Abduction**	257	**3.16** *± 1.52*	**2.34** *±1.37*	**4.67** *± 2.29*	**5.38** *±2.57*

id	content	Ratings (n)	Valence (M ± SD)		Arousal (M ± SD)	
			1-sec	5-sec	1-sec	5-sec
55	**Fight**	253	**2.79** ± *1.42*	**3.03** ±*1.50*	**4.94** ± *2.24*	**4.97** ±*2.24*
56	**Parade**	258	**4.24** ± *1.42*	**4.38** ±*1.45*	**3.29** ± *1.97*	**3.10** ±*1.86*
57	**Gun**	264	**3.73** ± *1.42*	**3.35** ±*1.54*	**3.97** ± *2.20*	**4.71** ±*2.37*
58	**Weapon**	254	**4.45** ± 1.29	**3.48** ±*1.46*	**3.40** ± *2.01*	**4.41** ±*2.16*
59	**Women Gun**	237	**4.02** ± *1.43*	**3.93** ±*1.45*	**4.40** ± *2.27*	**4.82** ±*2.26*
60	**Dead Body**	268	**2.79** ± *1.50*	**2.70** ±*1.48*	**4.39** ± *2.48*	**4.65** ±*2.52*
61	**Hostage**	233	**3.31** ± 1.55	**2.96** ±*1.49*	**4.47** ± *2.25*	**5.05** ±*2.32*
62	**Surface mining**	254	**4.47** ± *1.56*	**4.29** ±*1.59*	**2.90** ± *1.87*	**2.99** ±*1.87*
63	**Police**	248	**3.95** ± *1.37*	**4.06** ±*1.39*	**3.87** ± 2.07	**3.79** ±*2.06*
64	**Police**	227	**3.85** ± *1.33*	**3.60** ±*1.44*	**4.22** ± *2.09*	**4.48** ±*2.22*
65	**Deforestatio n**	239	**4.19** ± *1.50*	**3.65** ±*1.65*	**2.82** ± *1.87*	**3.07** ±*2.05*
66	**Pollution**	262	**4.13** ± *1.40*	**4.05** ±*1.48*	**3.14** ± *1.97*	**3.11** ±*1.99*
67	**Pollution**	252	**3.74** ± *1.53*	**2.98** ±*1.48*	**3.33** ± *1.90*	**4.07** ±*2.14*
68	**Mining**	251	**4.38** ± *1.20*	**4.41** ±*1.31*	**3.33** ± *1.88*	**3.20** ±*1.86*

id	content	Ratings (n)	Valence (M ± SD)		Arousal (M ± SD)	
			1-sec	5-sec	1-sec	5-sec
69	**Pollution**	241	**4.33** *± 1.37*	**4.24** *±1.45*	**2.86** *± 1.75*	**2.90** *±1.90*
70	**Crying**	249	**4.76** *± 1.25*	**4.31** *±1.28*	**2.50** *± 1.81*	**2.53** *±1.77*
71	**Shot**	245	**2.92** *± 1.42*	**2.29** *±1.39*	**4.32** *± 2.29*	**5.30** *±2.56*
72	**Threatend**	235	**3.34** *± 1.41*	**2.29** *±1.41*	**3.75** *± 1.97*	**5.72** *±2.57*
73	**Nazi**	265	**2.62** *± 1.50*	**2.02** *±1.31*	**4.61** *± 2.37*	**5.32** *±2.44*
74	**Crying**	280	**4.39** *± 1.25*	**4.02** *±1.35*	**2.66** ± 1.71	**2.78** *±1.73*
75	**Water body**	231	**3.88** *± 1.63*	**3.60** *±1.65*	**3.98** *± 2.20*	**4.21** *±2.33*
76	**Hospital**	265	**3.85** *± 1.27*	**3.54** *±1.35*	**3.28** *± 1.89*	**3.37** *±1.97*
77	**Hospital**	241	**3.13** *± 1.32*	**3.17** *±1.36*	**3.54** *± 2.07*	**3.55** *±2.09*
78	**Fight**	243	**2.90** *± 1.40*	**2.04** *±1.27*	**5.01** *± 2.27*	**5.90** *±2.60*
79	**Running**	268	**4.62** *± 1.40*	**3.82** *±1.46*	**4.08** *± 2.01*	**4.64** *±2.27*
80	**Attack**	262	**2.83** *± 1.35*	**2.42** *±1.34*	**4.92** *± 2.37*	**5.54** *±2.48*
81	**Ants**	273	**3.99** *± 1.51*	**3.99** *±1.66*	**3.88** *± 2.26*	**3.83** *±2.35*
82	**Chicken**	292	**2.67** *± 1.52*	**2.07** *±1.39*	**4.68** *± 2.35*	**5.29** *±2.49*

id	content	Ratings (n)	Valence (M ± SD)		Arousal (M ± SD)	
			1-sec	5-sec	1-sec	5-sec
83	**Chicken**	283	**3.32** *± 1.75*	**2.11** *±1.38*	**4.22** *± 2.30*	**5.42** *±2.59*
84	**Flood**	290	**4.66** *± 1.44*	**4.57** *±1.49*	**3.16** *± 1.89*	**3.37** *±2.02*
85	**Hitting**	317	**3.34** *± 1.44*	**2.86** *±1.54*	**4.49** *± 2.17*	**5.00** *±2.34*
86	**Threatend**	273	**3.94** *± 1.37*	**3.23** *±1.40*	**4.17** *± 2.19*	**4.76** *±2.29*
87	**Fight**	293	**2.36** *± 1.39*	**2.23** *±1.38*	**5.65** *± 2.49*	**5.87** *±2.49*
88	**Crying**	293	**3.82** *± 1.27*	**3.54** *±1.34*	**3.13** *± 1.88*	**3.31** *±1.90*
89	**Hostage**	278	**2.83** *± 1.40*	**2.73** *±1.34*	**4.84** *± 2.26*	**4.86** *±2.31*
90	**Abduction**	285	**3.47** *± 1.40*	**3.12** *±1.43*	**4.72** *± 2.22*	**5.16** *±2.31*
91	**Helicopter**	285	**4.27** *± 1.38*	**3.54** *±1.48*	**2.94** *± 1.90*	**3.52** *±2.01*
92	**Hostage**	296	**3.31** *± 1.43*	**2.24** *±1.27*	**4.47** *± 2.19*	**5.51** *±2.44*
93	**Dead Animal**	285	**3.04** *± 1.50*	**2.68** *±1.38*	**3.98** *± 2.18*	**4.25** *±2.23*
94	**Ambulance**	295	**4.57** *± 1.19*	**4.44** *±1.23*	**3.03** *± 1.85*	**3.25** *±1.91*
95	**Hostage**	289	**2.67** *± 1.35*	**2.50** *±1.47*	**5.20** *± 2.21*	**5.56** *±2.35*
96	**Grave**	304	**3.18** *± 1.37*	**3.12** *±1.40*	**3.04** *± 1.85*	**3.15** *±1.86*

id	content	Ratings (n)	Valence (M ± SD)		Arousal (M ± SD)	
			1-sec	5-sec	1-sec	5-sec
97	**Soldiers**	290	**4.57** *± 1.22*	**4.64** *±1.23*	**3.06** *± 1.87*	**2.93** *±1.80*
98	**Dead Boy**	300	**3.03** *± 1.46*	**2.78** *±1.46*	**3.37** *± 2.10*	**3.44** *±2.11*
99	**Hospital**	299	**3.42** *± 1.39*	**3.62** *±1.44*	**3.62** *± 2.06*	**3.64** *±2.08*
100	**Plastic ocean**	272	**3.66** *± 1.61*	**2.65** *±1.32*	**3.15** *± 1.90*	**3.51** *±2.11*
101*	**Elephant attack**	290	**5.23** *± 2.05*	**3.01** *±1.92*	**4.26** *± 2.18*	**5.64** *±2.26*
102	**Arrest**	315	**3.86** *± 1.30*	**4.11** *±1.43*	**4.18** *± 2.10*	**4.35** *±2.09*
103	**Arrest**	291	**3.83** *± 1.39*	**3.67** *±1.53*	**4.09** *± 2.09*	**4.20** *±2.24*
104	**Blood Animal**	286	**2.31** *± 1.40*	**2.05** *±1.34*	**5.59** *± 2.44*	**5.93** *±2.48*
105	**Car snow**	291	**4.25** *± 1.24*	**4.00** *±1.38*	**3.63** *± 2.09*	**4.60** *±2.29*
106	**Crying**	306	**3.10** *± 1.35*	**2.90** *±1.39*	**3.95** ± 2.18	**4.07** *±2.27*
107	**Women street**	289	**4.83** *± 1.18*	**4.33** *±1.31*	**2.88** *± 1.82*	**3.71** *±2.24*
108	**Attack**	298	**3.96** *± 1.31*	**3.58** *±1.33*	**4.98** *± 2.22*	**5.40** *±2.26*
109	**shooting**	281	**3.31** *± 1.54*	**2.98** *±1.55*	**5.02** *± 2.30*	**5.57** *±2.46*
110	**shooting**	280	**2.76** *± 1.26*	**2.54** *±1.37*	**5.04** *± 2.19*	**5.50** *±2.37*

id	content	Ratings (n)	Valence (M ± SD)		Arousal (M ± SD)	
			1-sec	5-sec	1-sec	5-sec
111	**Flood**	297	**2.69** ± 1.36	**2.28** ±*1.36*	**4.84** ± *2.38*	**5.20** ±*2.45*
112	**Fire**	265	**2.79** ± *1.40*	**2.70** ±*1.50*	**4.46** ± *2.31*	**4.38** ±*2.37*
113	**Snake**	285	**4.50** ± *1.70*	**4.64** ±*1.83*	**4.03** ± 2.39	**3.88** ±*2.33*
